# The Hippo–YAP Signaling as Guardian in the Pool of Intestinal Stem Cells

**DOI:** 10.3390/biomedicines8120560

**Published:** 2020-12-01

**Authors:** Yoojin Seo, So-Yeon Park, Hyung-Sik Kim, Jeong-Seok Nam

**Affiliations:** 1 Department of Life Science in Dentistry, School of Dentistry, Pusan National University, Yangsan 50612, Korea; amaicat24@naver.com; 2 Dental and Life Science Institute, Pusan National University, Yangsan 50612, Korea; 3 School of Life Sciences, Gwangju Institute of Science and Technology, Gwangju 61005, Korea; sypark0125@gist.ac.kr

**Keywords:** intestine, intestinal stem cells, Hippo, YAP, homeostasis, regeneration, tumorigenesis

## Abstract

Despite endogenous insults such as mechanical stress and danger signals derived from the microbiome, the intestine can maintain its homeostatic condition through continuous self-renewal of the crypt–villus axis. This extraordinarily rapid turnover of intestinal epithelium, known to be 3 to 5 days, can be achieved by dynamic regulation of intestinal stem cells (ISCs). The crypt base-located leucine-rich repeat-containing G-protein-coupled receptor 5-positive (Lgr5^+^) ISCs maintain intestinal integrity in the steady state. Under severe damage leading to the loss of conventional ISCs, quiescent stem cells and even differentiated cells can be reactivated into stem-cell-like cells with multi-potency and contribute to the reconstruction of the intestinal epithelium. This process requires fine-tuning of the various signaling pathways, including the Hippo–YAP system. In this review, we summarize recent advances in understanding the correlation between Hippo–YAP signaling and intestinal homeostasis, repair, and tumorigenesis, focusing specifically on ISC regulation.

## 1. Introduction

The small intestinal epithelium consists of single-layered, well-organized structures called the crypt–villus unit [1,2]. Intestinal stem cells (ISCs) and their niche-forming Paneth cells reside at the crypt base, and ISC-derived progenitors travel upwards from the crypt to the villus, consisting of a transit-amplifying (TA) zone. Eventually, most of the differentiated epithelial cells are found in the villi region, except for Paneth cells that migrate downward to support the ISCs [3,4]. No villus-like projection was observed in the colon; however, the colonial crypt also contains a Paneth-cell-like epithelial niche called deep secretory cells and stem cells, while differentiated progeny cells are distributed in the upper region as observed in the small intestine [5,6].

ISCs replenish all intestinal epithelial cells (IECs) throughout the intestinal tract. For instance, at least six different epithelial lineages comprising the small intestine can be derived from the leucine-rich repeat-containing G-protein-coupled receptor 5 (Lgr5)-positive ISC pool [7,8]. These lineage-committed cells are classified into two broad groups—absorptive cells (enterocytes and M cells) and secretory cells (Paneth cells, enteroendocrine cells, goblet cells, and tuft cells) [2]. Even under normal conditions, terminally-differentiated cells continue to migrate to the tip of the villi, where they undergo an apoptotic process called anoikis [9]. It is not surprising, therefore, that ISCs exhibit a profound cell division capacity with high telomerase activity to produce their progeny consistently and maintain their self-renewal [10]. In addition, the damaged intestinal epithelium can be recovered without Lgr5^+^ ISCs via cellular reprogramming of differentiated cells or reactivation of quiescent stem cells, implying that the superior regenerative potency of the intestine rather than other adult tissues is exerted by dynamic regulation of IECs in a context-dependent manner [11].

The development of cell-type-specific promoter-based lineage-tracing techniques and the emergence of single-cell RNA sequencing analysis provides an in-depth understanding of the characteristics of IECs and regulatory-signaling pathways [8,12,13]. Furthermore, the recently-introduced intestinal organoid culture system can successfully mimic the nature of IECs from expansion to differentiation in vitro, which provides more intuitive and reliable research techniques with high reproducibility compared to conventional 2D culture [14,15]. Owing to these technological advances, several key pathways and crucial factors composing the intestinal microenvironment and their functions have been described. Wnt-, Notch-, epithelial growth factor (EGF)-, and bone morphogenetic protein (BMP)-signaling pathways are regarded as canonical ISC niche pathways [16]. In addition, increasing evidence suggests the involvement of Hippo–YAP signaling, which plays an indispensable role in controlling organ growth during the developmental stage, in ISC regulation. Here, we summarize the latest perspectives on ISCs and recent studies explaining how the Hippo– YAP pathway can contribute to intestinal homeostasis, regeneration, and tumorigenesis.

## 2. The Characteristics of Steady-State and Injury-Associated ISCs

### 2.1. Crypt Base Columnar Cells (CBCs): Homeostatic ISCs

The presence of continuously-dividing stem cells in the crypt base was first described in the 1970s [17]. These cells were referred to as ‘CBCs’ since they are found in the crypt bottom at +1 to +3 positions between Paneth cells. In 2007, Barker et al. conducted a screening for gene expression patterns for Wnt targets within the crypt and suggested that Lgr5 is a unique CBC marker [8]. They labeled a pool of active cycling Lgr5^+^ ISCs for lineage tracing and found that CBCs are genuine stem cells that can replenish the entire crypt–villus axis in the intestinal tract. Lgr5 is a Wnt target gene and when it binds to its ligand, R-spondin, it is internalized with Wnt-degrading E3 ubiquitin ligase RNF43 and ZNRF3, which potentiate Wnt signaling [18]. CBCs are committed to differentiation when Wnt signaling is off; therefore, both Wnt ligand and R-spondins play crucial but non-overlapping roles in maintaining active self-renewal of ISCs [19]. Under homeostatic conditions, they undergo symmetric cell division with neutral competition within the crypt base [20,21], while CBCs pushed out of the niche lose their stemness and then enter the TA zone to be differentiated [22]. This journey to the villi is not a passive but an active process; indeed, migrating cells acquire the polarity towards the upside characterized by actin-related protein 2/3 (Arp2/3) complex-mediated protrusion formation [23]. These findings collectively provide valuable insights into how CBCs can maintain the integrity of the intestinal epithelium, one of the fastest self-renewing tissues in mammals [24].

### 2.2. Label-Retaining Cells (LRCs) or +4 Cells: Quiescent, Reserve ISCs?

**Figure 1:**
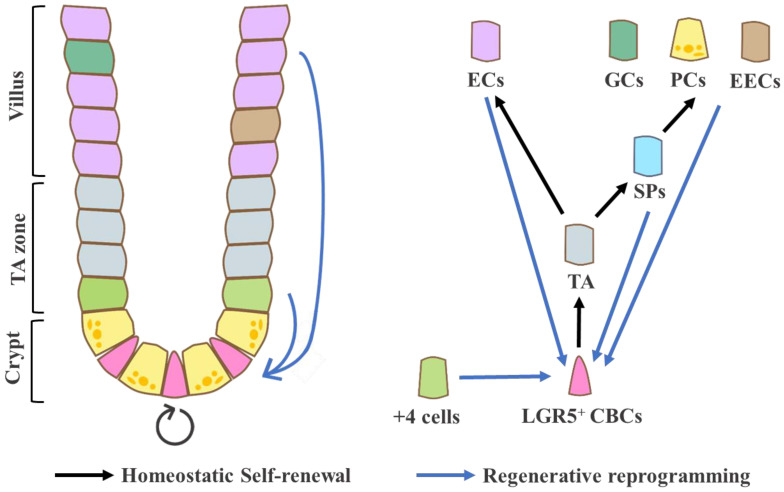
The intestinal crypt–villus structure and cell specification of intestinal stem cell (ISC)-differentiated intestinal epithelial cells (IECs) during homeostatic conditions (black arrow) and injury-induced cellular reprogramming (blue arrow). CBCs, crypt base columnar cells; TA, transit-amplifying cells; SPs, secretory precursors; ECs, enterocytes; GCs, goblet cells; PCs, Paneth cells; EECs, enteroendocrine cells.

The +4 cells are located between the CBC-Paneth cell-rich crypt zone and the TA zone, usually just above the uppermost Paneth cell [25]. Besides the location, the most distinctive feature of +4 cells compared to CBCs is their label-retaining property. As revealed by a double DNA labeling strategy using tritiated thymidine and bromodeoxyuridine, they are non- or slowly-dividing cells in homeostatic conditions [26], implying that +4 cells represent a ‘quiescent’ stem cell population [27]. Various candidates have been suggested as +4 cell markers, including Bmi1, Hopx, Tert, Sox9, and Lrig1 [28,29,30,31,32,33,34]. For instance, Bmi^+^ cells are less dependent on the Wnt pathway and resistant to radiation injury than CBCs [34]. In addition, they can be re-activated and give rise to IECs including Lgr5^+^ CBCs both in vivo and in vitro, confirming their multi-potency as stem cells [35]. However, since most of these markers are not restricted to +4 cells but are also expressed in other cells, including CBCs [36], the debate continues about the genuine marker for these label-retaining cells. Surprisingly, quiescence-associated genomic profiling can be acquired by secretory precursors and a subset of enteroendocrine cells during the injury and repair process [37,38], suggesting that the intestinal epithelium has a dynamic, heterogeneous pool of ‘potential’ or ‘reverse’ stem cells based on their cellular plasticity (Figure 1).

### 2.3. Damage-Induced ISCs: Reverse- or Revival Stem Cells (RSCs) and Reprogramming Cells

Despite the genetic ablation or injury-associated loss of CBCs, the intestinal epithelium can be completely regenerated; thus, many efforts have been made to define the alternative cellular source of conventional ISCs during the regenerative procedure using the lineage-tracing technique. Since the first evidence was presented in 2012 [39], secretory precursors have been one of the most reliable candidates for this phenomenon. Notch signaling plays pivotal roles in IEC differentiation and it functions as a determinant for lineage specification via a mechanism called ‘lateral inhibition’ [40]. Notch ligands Delta-like 1 and 4 (Dll1 and Dll4) are consistently expressed in Paneth cells and activate Notch signaling in adjacent cells to repress the expression of Math1, the major transcription factor for the secretory lineage differentiation [40,41]. In addition, Dll1-high cells in the TA zone function as secretory precursors for Paneth cells, goblet cells, and enteroendocrine cells, while no enterocytes are derived from Dll1-high cells [39]. Dll1-high cells can generate mature organoids in vitro with a supplement of extra Wnt and they can replenish whole IECs upon irradiation damage. Similar secretory progenitors for ISC substitutes are also found in the colon [42]. In this study, the authors reported that Lgr5^+^ ISC-depleted mice can be recovered from experimental colitis due to the contribution of Atoh1^+^ secretory progenitors to IEC regeneration. Considering their position, Dll1-high or Atoh1^+^ secretory precursors might belong to the LRC population. Indeed, LRCs share the transcriptional phenotype of secretory lineages including Mmp7, Kit, Chga, Gip, and Pax6 [37,43]. In addition, not only precursors but also terminally-differentiated mature IECs can re-enter the ISC-like status. These cells include enteroendocrine cells [38], Paneth cells [44,45], and even enterocytes [46]. Specifically, Tetteh et al. traced the fate of fully-differentiated Alpi^+^ enterocytes after the depletion of Lgr5^+^ cells and unexpectedly found that these cells migrated downwards to the crypt base and de-differentiated into multiple cell types of cells, including stem cells and secretory cells [46], implying the high flexibility of the intestinal epithelium during the repair process.

Collectively, it is now widely accepted that cellular reprogramming of differentiated cells into ISCs is one of the primary strategies for intestinal recovery; however, to date, the underlying molecular mechanisms that regulate cellular plasticity remain obscure. It is reported that these LRC-like secretory precursors are under the dynamic modulation of chromatin accessibility and that Lgr5^+^ ISC-associated signature genes become open chromatic states during the de-differentiation process [43]. Others have revealed that the intestinal repair process is accompanied by the acquisition of a fetal-like expression profile, represented by the emergence of stem cell antigen-1 (Sca-1)^+^ cells [47,48]. A recent study has also discovered a rare population among the crypt base expressing Clusterin (Clu), which is in a quiescence state under homeostatic conditions but reactivated upon injury to ‘revive’ the crypt [49]. Notably, cellular reprogramming towards primitive profiles is crucial for the initiation of the intestinal regeneration process, and Hippo– YAP signaling plays an indispensable role in this context.

## 3. The Hippo Pathway

### 3.1. Core Components of the Hippo Pathway

The Hippo pathway is an evolutionarily-conserved signaling pathway involved in various cellular functions. The pathway was initially described through genetic screening for tumor suppressors involved in regulating tissue growth in *Drosophila melanogaster*. In *Drosophila*, the core components of the Hippo pathway, including Hippo (Hpo) [50,51,52,53], Salvador (Sav) [54], Warts (Wts) [55], Mats [56], Yorkie (Yki) [57], and Scalloped (Sd) [58,59], coordinate tissue growth.

The core kinase cascade and downstream effectors of the Hippo pathway discovered in flies are highly conserved in mammals. The mammalian sterile 20-like 1 and 2 (Mst 1 and 2) are the first kinases of the mammalian Hippo pathway and are homologues of Hpo [51]. Activated Mst1/2 forms heterodimers with Sav1, a Sav homologue, to phosphorylate large tumor suppressor 1 and 2 (Lats 1/2, Wts homologues), as well as Mob1a/b (Mats homologues) [60]. Lats 1/2 activation by phosphorylation and the interaction with phosphorylated Mob1 directly phosphorylates Yes-associated protein 1 (YAP 1 or YAP, a Yki homolog) and a transcriptional co-activator with PDZ-binding motif (TAZ) to inhibit their activities [61,62,63]. The phosphorylation of YAP and TAZ by Lats 1/2 is a crucial reaction in the Hippo pathway, which results in the sequestration of YAP and TAZ in the cytoplasm as an inactivated state [64,65,66]. Unphosphorylated YAP and TAZ accumulate in the nucleus to form a complexes with DNA-binding transcription factor TEA domain family members 1–4 (TEAD 1–4, homologues of Sd). The YAP/TAZ and TEAD complex regulate the expression of target genes, associated with cell proliferation and organ growth [67,68,69,70]. Although TEAD family transcription factors are major partners for interaction with YAP/TAZ, other transcription factors including p73, runt-related transcription factor (Runx) 1/2, Smad, T-box transcription factor 5 (Tbx5), and paired box gene 3 (Pax3), have been reported to interact with YAP/TAZ to modulate the expression of diverse genes [71,72,73,74].

### 3.2. Upstream and Cross-Talk Signaling of the Hippo Pathway

The Hippo pathway is regulated by variable upstream inputs or cross talk with other signaling pathways. So far, the Hippo pathway does not have specific receptors or relevant ligands, unlike other well-known classical signaling pathways. Instead, upstream components involved in polarity, morphology, and adhesion of cells have been reported to regulate the Hippo pathway [75,76,77,78,79,80,81]. The Hippo pathway is activated in responding to mechanical cellular stresses, changes in nutrients, or the adhesion between cell-to-cell or cell-to-extracellular matrix (ECM), rather than specific signaling ligands [82,83,84].

Most cells show polarity, represented by apical–basal polarity and planar cell polarity observed in epithelial cells. Both types of polarity can regulate the Hippo pathway. The apical–basal cell polarity is established by protein complexes that make up cell–cell junctions, including adherent and tight junctions. In the *Drosophila* epithelium, apical membrane-localized Merlin (Mer), Expanded (Ex), and kidney- and brain-expressed protein (Kibra) constitute a complex to cooperatively activate the Hippo pathway by binding to Sav, Hpo, and Wts [85,86,87]. It also conserves this mechanism of the apical protein complex in mammals. The complex of neurofibromin2 (Nf2, a Mer homologue), Kibra- and FERM-domain-containing 6 (FRDM6, an Ex homologue) suppress the activity of YAP [88]. Nf2 is a gene reported in the Hippo pathway which is mutated in cancers of the central nervous system [89]. Planar cell polarity, the positional and directional polarity of cells in a layer, can also modulate the Hippo pathway. In *Drosophila*, the transmembrane cadherins Fat (Ft) and Dachsous (Ds) form a complex and activate the Hippo pathway through the induction of Wts degradation [90,91]. Although mammalian homologues for Ft (Fat 1–4) and Ds (Hchs 1/2) are present, the action of these homologues in the Hippo pathway is less clear and further studies are required [92,93].

Several groups have demonstrated that structural proteins of tight junction and adherens junctions can interact with YAP. A tight junction protein, angiomotin (Amot) can inhibit YAP by physically interacting and transferring YAP from the cytoplasm to tight junctions or actin filaments, lessening the phosphorylation status of YAP [94]. In addition, Amot proteins can also activate Lats 1/2, resulting in the phosphorylation of YAP [95]. Another tight junction protein, Zonula occludens-2 (ZO-2), was shown to localize YAP to the nucleus [96] and TAZ to tight junctions [97]. A component of the adherens junction, α-catenin, was reported to form a complex with phosphorylated YAP, which inhibits YAP activity [98].

YAP activity is also sensitive to mechanical cues from neighboring cells or the ECM. Indeed, ECM stiffness, cell tension, cell spreading, and cell attachment or detachment were proven to modulate the Hippo pathway [76,80,81]. For instance, mechanical stresses that are caused by cell growth on a surface with high stiffness or exposure to shear stress can induce the nuclear translocation of YAP and TAZ [76,80,99,100], whereas cell detachment from the ECM triggers the export of YAP and TAZ from the nucleus [81]. Signaling cues generated by integrin complexes at adhesion sites responding to mechanical ECM properties induce changes in intracellular actin cytoskeleton dynamics, resulting in the regulation of the Hippo pathway [101,102].

Recently, several studies have suggested that extracellular ligands can regulate the activity of YAP and TAZ for G-protein-coupled receptors (GPCRs). GPCR signaling can either activate or inactivate YAP and TAZ, in a manner dependent on the class of G proteins. For instance, the activation of Gα_12/13_, Gα_q/11_, or Gα_i/o_ by coupled ligands including lysophosphatidic acid, sphingosine 1-phosphate, and thrombin receptor agonists can stimulate YAP and TAZ, whereas Gα_s_ activation with glucagon and epinephrine can suppress YAP and TAZ [103,104,105,106]. It has been reported that Rho GTPase and actin cytoskeleton are involved in regulating YAP and TAZ by diverse GPCR receptor signaling. In general, Lats 1/2 mediate the activity of Rho GTPase and actin cytoskeleton in response to GPCR ligands to regulate YAP and TAZ.

Moreover, other signaling pathways such as Wnt, Notch, Sonic hedgehog (Shh), and epithelial growth factor receptor (Egfr)/Kras, have been shown to cross talk with the Hippo pathway [107,108,109]. Discovery of connections between the Wnt and Hippo pathways improved the understanding of the homeostatic mechanism of organs [89]. Glycogen synthase 3 (GSK3), a key enzyme in Wnt signaling repressed by Wnt, is reported to directly phosphorylate and degrade TAZ [110]. TAZ also interacts with β-catenin, a core component in the transduction of Wnt signals, and degraded together when β-catenin is phosphorylated by GSK3 [111]. In addition, the activation of the Hippo pathway has been shown to inhibit Wnt/β-catenin signaling through the interaction between TAZ and Disheveled (DVL), an essential scaffold protein in the Wnt pathway [112]. Several studies have demonstrated that the regulation of the Wnt pathway by the Hippo pathway depends on Hippo signaling. That is, YAP and TAZ in the cytoplasm can directly suppress the nuclear translocation of β-catenin [113]. On the other hand, YAP in the nucleus can increase the expression of Wnt target genes by boosting β-catenin activation [114,115].

The Hippo pathway also has cross talk with Notch and Shh signaling pathways. Deletion of Mst 1/2 or overexpression of YAP has been shown to induce Notch activation [116,117]. In addition, YAP is involved in the expression of Jagged-1, a Notch ligand [118]. A study suggested that YAP can directly induce the expression of Gli2, a downstream effector in the Shh-signaling pathway. Conversely, YAP has been reported to be crucial for Shh-mediated tumorigenesis in medulobalstoma [119].

Recent studies have shown that YAP protein can regulate the Egfr/Kras signaling pathway. For example, it was found that EGFR-activated Ras during tumorigenesis up-regulates the level of cytoplasmic YAP protein and unphosphorylated YAP, which in turn, can induce Egfr transcription to create a positive feedback loop [107]. Moreover, interaction between YAP and Egfr was shown to be associated with the progression of esophageal cancer in patients treated with Egfr inhibitor [120]. Another recent study demonstrated that a feedback loop generated by YAP and Egfr can affect tumorigenesis and progression of ovarian cancer [121].

## 4. The Contribution of Hippo–YAP Signaling Pathway in the Intestine

### 4.1. Intestinal Homeostasis

As observed in other organs, the Hippo signaling pathway is indispensable during the steady state of the mammalian intestinal epithelium. IEC-specific Mst1/Mst2 double knockout mice exhibit a short life span (13 weeks in median) with severe wasting signs and disorganized crypt–villus structure due to the hyperplasia of ISCs and undifferentiated cells are found in their intestinal tract, leading to adenoma formation [117]. A similar phenotype is observed in the Sav1-depleted intestine; upon the loss of Sav1, crypts become enlarged and disoriented which in turn develops into the colonic polyps [122]. In both papers, the authors have proven that the tumorigenic and hyper-proliferative features of the Hippo pathway-deficient intestine can be reversed by blocking YAP signaling, implying that consistent suppression of YAP activity via active Hippo pathway is required to maintain intestinal homeostasis. Indeed, the physiological role of Hippo signaling as a negative regulator of YAP is also confirmed by in vivo delivery of Lats1/2 siRNA, which leads to massive crypt proliferation and goblet cell differentiation due to YAP overabundance [123].

The nuclear accumulation of YAP in the IECs is dynamically regulated along the crypt–villus axis. In general, a high level of nuclear YAP is detected at the ISC-rich crypt base (except for Paneth cells), while it tends to be translocated into the cytoplasm and diminished in the upper region of the villi [116,122,124]. It has been clearly shown that stable activation of intestinal YAP either by disruption of the upstream inhibitor Hippo signaling or by enhancing the nuclear translocation drives massive ISC proliferation without proper differentiation and maturation processes [116,117,122,125]. Camargo et al. indicated that systemic YAP1 activation leads to the most dramatic change in the intestinal epithelium, where most secretory cells such as Paneth cells and goblet cells are absent, and undifferentiated progenitors are profoundly expanded instead [116]. Notably, treatment with the γ-secretase inhibitor, dipenzazepine, can partially reverse the intestinal dysplasia caused by YAP activation, suggesting a mediating role of Notch signaling in this context, as reported previously [117]. Besides Hippo signaling, the YAP pathway interacts with other key signaling pathways such as Wnt and Notch signaling, which interfere with intestinal homeostasis [126]. In addition, the tumor suppressor protein kinase C ζ (PKCζ) is also involved in phosphorylation-mediated YAP inhibition in IECs in the steady-state, given that deletion of PKCζ induces YAP activation, resulting in the increment of self-renewal potential of Lgr5^+^ ISCs in both the in vivo and in vitro organoid culture systems [127]. In contrast, others reported that IEC-specific YAP induction resulted in a significant loss of proliferating crypts with decreased expression of typical CBC markers such as Olfm4 [128]. Both microarray data and gene set enrichment analysis showed that signature CBC genes, mainly expressed by active Wnt pathway, are downregulated in the YAP-activated crypts compared to normal ones. Of note, the necessity of YAP signaling in the homeostatic state remains somewhat controversial. Several studies have reported that no significant changes in cell proliferation and differentiation are observed in the intestinal epithelium of YAP-OFF mutants during steady state [117,122,124]; however, Imajo et al. showed that double knockdown of YAP/TAZ using intestine-specific gene transfer technique suppresses crypt cell proliferation via modulation of Wnt signaling [123]. They further revealed that sufficient YAP activity is required for goblet-cell differentiation as YAP/TAZ cooperate with one of the goblet-cell-specification transcription factors, Klf4. In addition, intestinal organoid formation efficiency varies depending on the stiffness of polyethylene glycol (PEG) hydrogels and the mechanosensing property of YAP is involved in this phenomenon [129]. Organoid-forming efficiency is enhanced in intermediate (1.3 kPa)-hardened matrices, where YAP is predominantly localized in the nuclear fraction of ISCs. On the other hand, blocking YAP activity or growth in the soft gel, which induces cytoplasmic translocation of YAP, leads to growth arrest of the intestinal organoid.

These contradicting observations regarding the role of YAP in intestinal homeostasis might be derived from the differences between YAP activation (systemic vs. IEC-specific), the complexity of ISC-regulating signaling pathways, the possible contribution of TAZ for YAP activity [112,128], and the duration of YAP induction or suppression. Notably, both prolonged activation and inhibition of YAP dampen intestinal organoid formation [130]. In this study, Serra et al. investigated the cellular dynamics and signaling requirements in Lgr5^+^ single-cell-derived intestinal organoid development. They compared the trajectory of ISC dynamics between normal budding organoids and spheroid-like enterocysts in a time-dependent manner and found that YAP1 and its target genes are transiently activated for the induction of a symmetry-breaking process. Interestingly, homogenous YAP activation prevents, as observed in the constant YAP-OFF state, the formation of budding structure, and further maturation of the organoid. They further revealed that uneven YAP activity induces Paneth cell generation by generating differences in cell-to-cell availability for Notch ligand Dll1, suggesting the contribution of YAP activity to the formation of an epithelial niche.

### 4.2. Intestinal Repair and Regeneration

To study the nature of intestinal regeneration, various experimental conditions such as irradiation exposure, chemotherapeutic 5-Fluorouracil injection, and administration of colitogenic agent dextran sodium sulfate (DSS) or 2,4,6-trinitrobenzene sulfonic acid (TNBS) are used [131,132,133]. Although the precise mechanism differs from each other, these treatments immediately lead to the enormous death of crypt cells, especially fast-cycling homeostatic Lgr5^+^ CBCs, followed by a robust regeneration.

To date, growing evidence has proven the involvement of Hippo and YAP signaling in tissue regeneration [134]. In general, proper YAP activation is essential for the intestinal repair process. After exposure to irradiation, nuclear translocation as well as the total expression level of YAP around the crypt area is greatly increased compared to the homeostatic condition [128,135]. In this context, YAP activity seems to downregulate the Wnt signaling and this transient inhibition is important to start regeneration properly since hyper-activation of the Wnt pathway either by loss of YAP or excessive R-spondin treatment induces the uncontrolled proliferation of crypt cells which finally leads to apoptosis and stem cell exhaustion [128,136]. Gregorieff et al. demonstrated the underlying mechanism for the YAP-dependent repair process upon irradiation using conditional YAP-OFF mice [135]. In this work, YAP impedes Wnt signaling not only to prevent the loss of the ISC pool but also to decrease ectopic Paneth cell differentiation. It was also revealed that the YAP-dependent induction of EGF receptor (Egfr) ligands is prominent during the regenerative process and in vitro treatment of Epiregulin can rescue the defect of YAP-deficient intestinal organoid formation, indicating the mediating- or compensatory role of Egfr signaling in YAP-associated intestinal regeneration. YAP activation is also required in the recovery of DSS- or TNBS-induced colitis models [122,137,138]. Notably, Hippo and YAP signaling might be implicated in the pathogenesis of inflammatory bowel disease (IBD) [137,138,139]. A link between intestinal inflammation and regeneration has been suggested by demonstrating that one of pro-inflammatory cytokine Interleukin-6 (IL-6) and its co-receptor gp130 contribute to stable YAP activation via Src family kinase (SFK) signaling [137]. The constant activation of the gp130 pathway can ameliorate the symptoms of DSS-induced colitis models, whereas IEC-specific ablation of YAP or treatment with the SFK pathway inhibitor offset the protective effects. Intestinal inflammation and the immune system might be involved in the regulation of Yap signaling since ablation of group 3 innate lymphoid cells (ILC3s) in the intestine impeded Yap-mediated regeneration, although the precise mechanism is under investigation [140]. Meanwhile, as observed in the homeostatic condition, persistent YAP activation can interfere with the regenerative process. Focusing on the fact that YAP expression is up-regulated in the IEC of IBD patients, the therapeutic effect of YAP-targeting microRNA miR-590-5p as well as YAP inhibitor verteporfin in the TNBS model was investigated [139]. However, considering the importance of early YAP activation as a ‘switch’ for the repair process, appropriate timing and duration for YAP suppression should be determined for the practical consideration.

During epithelial regeneration, cellular reprogramming such as cell fate conversion and de-differentiation occurs under the control of Hippo and YAP signaling. First, lineage-tracing analysis of Lyz^+^ cells in the crypt revealed that mature Paneth cells re-enter the cell cycle, divide actively, and re-differentiate into other cell types after irradiation [45]. These irradiated Paneth cells lose typical Paneth cell markers and acquire ISC-associated gene signatures. Hence, Paneth cells isolated from irradiated mice can generate organoids as Lgr5^+^ ISCs, confirming their clonogenic potential. Importantly, distinctive nuclear translocation of YAP precedes this process, implying its involvement in Notch-signaling-dependent reprogramming of Paneth cells. Indeed, a simple increment of Wnt signaling achieved by constitutive β-catenin induction cannot reproduce this phenomenon, but forced expression of the Notch signaling activator NICD1 induces de-differentiation of Paneth cells. Meanwhile, the role of YAP activation in the emergence of repairing-associated cells has been suggested [47]. After the DSS treatment, the reprogrammed repairing epithelium develops and persists during the regenerative period. In terms of transcriptional profile, repairing-associated epithelium resembles fetal epithelial program—for instance, it contains Sca1/Ly6a-expressing cells, which are usually found in fetal colonic epithelium. Interestingly, the extracellular matrix (ECM) composition of repairing epithelium is also altered from the homeostatic epithelium and integrin-focal adhesion kinase (FAK) signaling is profoundly up-regulated along with enhanced YAP activation. YAP–FAK-pathway mediated ECM remodeling is critical for epithelial regeneration given that FAK inhibitor-treated- or YAP/TAZ double knockdown mice cannot develop repairing epithelium upon DSS exposure. Similar to these observations, Ayyaz et al. recently discovered the subset of reprogrammed cells, so-called RSCs, as described in the Section 2.3, that contribute to the rehabilitation of damaged crypts in the absence of Lgr5+ ISCs [49]. Using a delicate single-cell RNA sequencing technique, they investigated the difference in cellular composition between normal- and irradiated crypts and revealed the Clu-expressing unique cell population. Clu^+^ cells hardly proliferate or differentiate under homeostasis; during the repair phase, on the other hand, they replace lost CBCs and re-constitute the entire epithelial structure as shown by crypt–villus ribbon formation. Strikingly, the gene signature of the Clu^+^ cluster of the damaged crypt is similar to the YAP-associated gene signature [135] and Lats1/2 double deletion induces the ectopic appearance of Clu+ cells even in the homeostatic condition, indicating that RSC generation depends on YAP activation.

In addition, one of the arachidonic acid derivatives prostaglandin E_2_ (PGE_2_) is regarded as a master regulator of YAP-induced intestinal cellular reprogramming. The therapeutic impact of PGE_2_ on intestinal repair and regeneration has been well established in various experimental models [141,142,143,144,145]. Kim et al. further elucidated the downstream of PGE_2_-mediated epithelial regeneration and that YAP plays a major role in this context [146]. First, the authors confirmed the contribution of PGE_2_ to the recovery of DSS-induced colitis by showing the increased epithelial regeneration of PGE_2_-degrading enzyme 15-PGDH-null mice. Upon binding the EP4 receptor, PGE_2_ increases the transcription of YAP and their target genes via the cyclic adenosine monophosphate response element-binding protein (CREB) pathway. Intact YAP signaling is critical for PGE_2_-derived therapeutic effect against DSS-induced colitis because either the deletion of one copy or complete loss of YAP in 15-PGDH-null mice significantly reduces regenerative efficacy. A recent study provides a more comprehensive and detailed perspective on how the PGE_2_–YAP axis regulates intestinal regeneration [147]. Single-cell RNA sequencing analysis of mouse intestinal mesenchyme reveals a subset of fibroblasts near the crypt base expressing cyclooxygenase-2 (Cox-2)-encoding gene Ptgs2, which provides PGE_2_ to the intestinal epithelium. These fibroblasts, named as rare pericryptal Ptgs2-expressing fibroblasts (RPPFs) by the authors, support the generation of Ly6a^+^ RSCs in the organoid culture system through the EP4 receptor. In line with the previous observation [146], PGE_2_-EP4 signaling turns on the YAP pathway via nuclear translocation and, in turn, induces YAP-associated transcriptional program including RSC signature genes such as Clu and Ly6a in organoids. Indeed, ISCs isolated from the YAP-deficient intestine do not respond to PGE_2_ treatment and fail to acquire an RSC-like phenotype. Collectively, these results indicate the critical role of YAP signaling in PGE_2_-induced cellular reprogramming during intestinal regeneration.

### 4.3. Intestinal Tumorigenesis

During inflammation or regeneration in the intestine, genetic mutations can occur and lead to malignant transformation [148]. Although the most frequent mutations in colorectal cancer (CRC) involve adenomatous polyposis coli (APC) gene and dysregulation of β-catenin signaling, the Hippo pathway has been found to contribute to CRC tumorigenesis [149,150,151]. Several mechanisms of CRC tumorigenesis regulation by the Hippo pathway have been discovered using intestinal epithelium-specific conditional knockout mice lacking Hippo pathway components, including Mst1, Mst2, Sav1, and YAP. Mice with conditional double knockout of Mst1 and Mst2 in the intestinal epithelium were reported to develop adenomas in the colon by 13 weeks of age via activated Wnt and Notch signaling followed by YAP protein up-regulation [117]. Moreover, Sav1 knockout mice were shown to develop colonic polyps at 13 months, similar to sessile serrated polyps observed in human lesions and chemically-induced intestinal injury further aggravated the tumorigenicity of these mice. The study also proved that this effect was YAP -dependent, given that mice with intestinal epithelium-specific knockout of Sav1 with YAP did not exhibit the formation of colonic polyps [122].

The oncogenic characteristics of YAP and TAZ in CRCs have also been supported by the results of analyzing patient tumor samples or human CRC cell lines. Indeed, the expression of YAP and TAZ was reported to have a positive correlation with poor prognosis in CRC patients, implying that the levels of YAP and TAZ might be prognostic indicators for CRC [117,122,152,153,154]. Moreover, YAP has been demonstrated to promote CRC resistance to chemotherapy, as well as CRC relapse [155]. Studies with human CRC cell lines have shown that inhibition of YAP expression remarkably decreased the proliferation and metastasis of CRCs, whereas overexpression of YAP accelerated the rate of cell proliferation [117,153]. Similarly, TAZ inhibition also led to a decrease in CRC proliferation [156].

As explained in the previous section, the Hippo pathway interacts with the Wnt/β-catenin signaling pathway, which plays a crucial role in the initiation of CRC. Loss-of-function mutation in APC gene has been frequently observed in CRCs [151]. APC mutation promotes CRC initiation through the activation of β-catenin and transcription-factor-4 (TCF4) complex which targets tumor progression-promoting genes, such as Myc, cyclin D1, and matrix metallopeptidase 7 (MMP7) [157,158,159,160,161]. Interestingly, several in vitro and in vivo studies have revealed that YAP and TAZ are activated in APC-deficient cells [111,124,162]. Moreover, intestine-specific knockout of Apc along with either YAP or TAZ did not exhibit intestinal hyperplasia [111,124]. Mechanisms for YAP/TAZ regulation by APC have been proposed. A study by Azzolin et al. showed the β-catenin destruction complex, a multiprotein complex including the tumor suppressors axin and APC, sequester YAP and TAZ in cytoplasm when Wnt signaling is off, whereas β-catenin, YAP, and TAZ are separated from the destruction complex and activated when Wnt signaling is activated by APC depletion [124]. Another study discovered the β-catenin destruction-complex-independent mechanism of YAP and TAZ regulation by APC. That is, APC promotes the activation of Lats by serving as a scaffold protein and APC loss results in the inactivation of the Hippo pathway [162]. β-catenin can also form a complex with YAP and Tbx5, which is essential for the survival of colon cancer driven by β-catenin through the induction of anti-apoptotic gene expression [115]. In addition to Wnt signaling, c-Jun N-terminal kinase (JNK) signaling has also been reported as an upstream regulator of the Hippo pathway in CRC. For instance, JNK activation in *Drosophila* was found to trigger the nuclear translocation of Yki, which further activates JAK/STAT signaling to increase cell proliferation [163]. However, the mechanistic correlation between JNK and the Hippo pathway has not been elucidated in mammals. Therefore, further studies are required to fully elucidate the role of the Hippo pathway along with other signaling pathways in the initiation and progression of CRCs.

## 5. Summary and Future Perspectives

**Figure 2:**
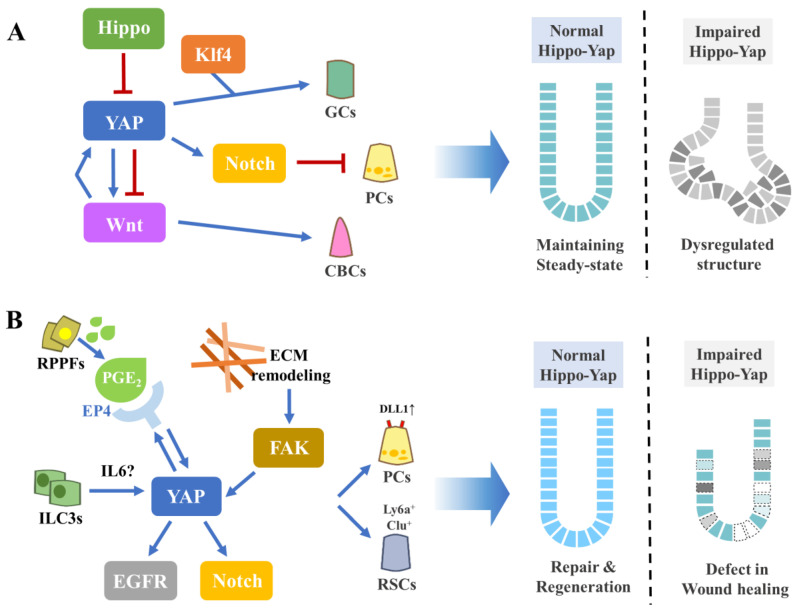
The Hippo–YAP signaling in intestinal homeostasis, regeneration, and tumorigenesis. (**A**) In the steady state, Hippo constantly suppresses the YAP signaling to maintain the crypt–villus integrity. (**B**) endogenous- and exogenous insults such as inflammation and irradiation induce PGE_2_ signaling or ECM remodeling, which in turn activates YAP signaling and initiates the cellular reprogramming process for the regeneration.

The intestinal epithelium exhibits a superior self-renewal potency based on stem-cell plasticity, and recent findings strongly imply the involvement of the Hippo–YAP pathway in this phenomenon. In the homeostatic intestine, active Hippo signaling is required to suppress YAP activity and in turn prevent uncontrolled overgrowth of the crypt. On the contrary, YAP signaling must be activated to induce the regeneration-associated alternative stem cells upon injury and dysregulated, and constant-activation of YAP leads to tumor initiation. In addition, the Hippo–YAP pathway seems to regulate the lineage specification of IECs, since YAP activation prevents Paneth cell induction while it drives Goblet cell differentiation. Depending on the context, Hippo–YAP signaling is involved in various pathways such as Wnt, Notch, and EGF. Inflammatory signals as well as alterations in the mechanical force due to ECM remodeling also regulate Hippo–YAP signaling in the intestinal epithelium (Figure 2).

Since the Hippo–YAP pathway can intervene in both intestinal regeneration and tumorigenesis, several attempts have been conducted to evaluate the therapeutic potential of Hippo–YAP modulatory drugs in these context. For instance, targeting the Hippo pathway by small molecule could stimulate the nuclear accumulation of YAP, which in turn promoted the intestinal repair process [164]. In this work, the authors discovered a highly-potent, selective MST1/2 inhibitor XMU-MP-1 via high-throughput screening. XMU-MP-1 abrogated the phosphorylation of MOB1, LATS1/2, and YAP both in vitro and in vivo, indicating that XMU-XP-1 can suppress the MST1/2 signaling cascade. Notably, daily administration of XMU-XP-1 provided some protection against DSS-induced colitis symptoms owing to the up-regulation of IEC proliferation. On the contrary, pharmacological inhibition of the YAP pathway is regarded as anti-cancer medication due to its pro-oncogenic property. Given that YAP/TEAD binding is required to activate the YAP-dependent downstream signaling, the anti-cancer role of small molecules targeting this interaction has been investigated. One of the Vestigial-like (VGLL) family of transcriptional cofactors, VGLL4, competes with YAP for pairing with TEAD and its synthetic peptide can suppress YAP activity, leading to gastric cancer inhibition [165]. Treatment with verterporfin, another well-described inhibitor of YAP/TEAD interaction [166], also suppressed the cancer-stem-cell-associated characteristics of gastric cancer cell line and inhibited tumor growth in xenograft model [167]. These studies collectively suggest the therapeutic potential of targeting the Hippo–YAP pathway in various circumstances; however, since both the improved regenerative capacity and oncogenic potential are correlated with YAP activation, the duration as well as the reversibility of pharmacological action should be carefully evaluated prior to the clinical application of Hippo/YAP modulators.

Although our understanding of both physiological and pathological roles of Hippo–YAP signaling in the intestine has been developed considerably, several questions remain to be addressed. There are still controversial observations regarding the impact of YAP activity in the homeostasis as well as disease progression. For instance, YAP activity is highest in the intestinal crypt and lowest in the villi, given the nuclear localization pattern of YAP; however, YAP signaling must be suppressed to control ISC proliferation. In addition, both consistent activation and suppression of YAP signaling impair crypt integrity. Hence, complicated cross talk with other pathways and how the duration and/or timing of YAP activation is regulated should be studied in detail. Finally, the possible contribution of Hippo–YAP signaling in the mesenchymal niche or other compartments of the intestinal microenvironment should be elucidated. Indeed, a recent work indicated the detrimental action of macrophage-derived YAP during IBD development [168]. Therefore, future research on lineage-specific action of Hippo–YAP signaling and their impact on the intestine would provide a more comprehensive view of intestinal homeostasis and disease.
